# Ultrasonic and Thermal Pretreatments on Anaerobic Digestion of Petrochemical Sludge: Dewaterability and Degradation of PAHs

**DOI:** 10.1371/journal.pone.0136162

**Published:** 2015-09-01

**Authors:** Jun Zhou, Weizhong Xu, Jonathan W. C. Wong, Xiaoyu Yong, Binghua Yan, Xueying Zhang, Honghua Jia

**Affiliations:** 1 College of Biotechnology and Pharmaceutical Engineering, Nanjing Tech University, Nanjing, China; 2 College of Environment, Nanjing Tech University, Nanjing, China; 3 Sino-forest Applied research Centre for pearl river delta Environment, Department of Biology, Hong Kong Baptist University, Hong Kong Special Administrative Region, China; 4 Lab of Waste Valorisation and Water Reuse, Qingdao Institute of Bioenergy and Bio process Technology, Chinese Academy of Sciences, Qingdao, China; Free University of Bozen/Bolzano, ITALY

## Abstract

Effects of different pretreatment methods on sludge dewaterability and polycyclic aromatic hydrocarbons (PAHs) degradation during petrochemical sludge anaerobic digestion were studied. Results showed that the total biogas production volume in the thermal pretreatment system was 4 and 5 times higher than that in the ultrasound pretreatment and in the control system, and the corresponding volatile solid removal efficiencies reached 28%, 15%, and 8%. Phenanthrene, paranaphthalene, fluoranthene, benzofluoranthene, and benzopyrene removal rates reached 43.3%, 55.5%, 30.6%, 42.9%, and 41.7%, respectively, in the thermal pretreatment system, which were much higher than those in the ultrasound pretreatment and in the control system. Moreover, capillary suction time (CST) of sludge increased after pretreatment, and then reduced after 20 days of anaerobic digestion, indicating that sludge dewaterability was greatly improved after anaerobic digestion. The decrease of protein and polysaccharide in the sludge could improve sludge dewaterability during petrochemical sludge anaerobic digestion. This study suggested that thermal pretreatment might be a promising enhancement method for petrochemical sludge solubilization, thus contributing to degradation of the PAHs, biogas production, and improvement of dewaterability during petrochemical sludge anaerobic digestion.

## Introduction

Petrochemical sludge is generated during petrochemical wastewater treatment in the petrochemical plants. It is mainly composed of waste oil, decomposable organics, polycyclic aromatic hydrocarbons (PAHs), pathogens, and mineral particles [1–2]. Consequently, improper treatment of this type of sludge may lead to accumulation of PAHs and pathogens along the food chain and may present potential threats to animal and human health. Therefore, better petrochemical sludge processing and utilization approaches should be explored.

Anaerobic digestion is usually used for the treatment of the municipal waste activated sludge. This treatment reduces the solid content, degrades organic pollutions, eliminates pathogenic microorganisms, and produces biogas, which can be used as a renewable energy. However, the concentrations of waste oil or PAHs in sludge may strongly inhibit the growth of anaerobic microorganisms and thus causing substantial delays in anaerobic digestion processes [3–4]. Consequently, the relevant researches about petrochemical sludge anaerobic digestion were very limited until now. On the other hand, the hydrolysis process is identified as the rate-limiting step in the process of producing methane because carbohydrates, proteins, and lipids, as well as soluble inert materials, are wrapped in sludge flocs structure and microbial cell walls, resulting in the difficult release of organic matters [5–6]. For this reason, to enhance the hydrolysis rate and the digestion performance, various pretreatment methods, such as mechanical [6], chemical [7–8], thermal [9], and enzymatic [10], have been applied to enhance the sludge solublization efficiency and subsequent improve anaerobic digestion performance based on different mechanisms. However, based on literature search, limited information could be found regarding the impact of different pretreatments on petrochemical sludge.

In addition, petrochemical sludge is usually treated as hazardous materials in many countries for its composition of waste oil and high concentration of PAHs. Therefore, improving the dewaterability of petrochemical sludge is very important to reducing sludge bulk and improving its handling properties. Extracellular polymeric substances (EPS) are believed to play an important role in the dewaterability of sludges [11–14]. Indeed, Houghton et al. [14] proposed that the decrease of sludge EPS content could make the sludge dewater more easily. Meanwhile, Yu et al. [15] and Neyens et al. [16] found that the composition and properties of EPS are more important in governing sludge dewaterability. The distribution and composition of sludge EPS would substantially change after sludge treated by different pretreatment methods [16–17], and then the sludge EPS would also change during the anaerobic digestion [18]. By contrast, limited information is available on the relationship between the variation of EPS and sludge dewaterability during petrochemical sludge anaerobic digestion with different pretreatments. Knowledge on the fate of PAHs during petrochemical sludge anaerobic digestion with different pretreatments is also poor.

Therefore, the objectives of the present study are to (1) evaluate the impact of different pretreatments on the improvement of solid reduction and biogas production; (2) elucidate the relationship between the content and composition of sludge EPS and sludge dewaterability during the petrochemical sludge anaerobic digestion process; and (3) investigate the biodegradation efficiency of the PAHs of petrochemical sludge during anaerobic digestion by different pretreatments.

## Materials and Methods

### Ethics statement

No specific permits were required for the described field studies and no specific permissions were required for these locations. The location is not privately-owned or protected in any way.

### Sludge sample and its characteristics

Petrochemical sludge was collected from the Yangzi petrochemical wastewater treatment plant and stored in a cold room at 4°C until use. The pH of wastewater sludge was measured immediately while solid content was measured by oven drying at 105°C. Dried sludge samples were analyzed for volatile solid (VS), total N, total P, as well as total concentrations of selected metals according to the standard methods [19]. Table 1 shows the selected physicochemical properties of the sludge.

**Table 1:** Physicochemical characteristics of petrochemical sludge (Values are on dry weight basis).

Parameters	Value
pH	7.28±0.02
Total solid (%)	2.28±0.02
Volatile solids (VS) content (%)	31.67±0.11
Total P (%)	0.50±0.03
Total N (%)	6.04±0.01
Zn (mg/kg)	392±11
Cu (mg/kg)	10.6±1.2
Cr (mg/kg)	9±0.26

### Pretreatment

Ultrasound and thermal methods were selected as the sludge pretreatment methods in this study. Ultrasonication pretreatment was performed using a Sartorius Labsonic-P Brandlab-scale sonicator, and petrochemical sludge was pretreated by ultrasound at 20 kHz and 480 W for 20 min [15]. Thermal pretreatment was conducted at temperature 130°C for 20 min in an autoclave [20]. After pretreatment, all treated sludge samples were stored at 4°C before anaerobic digestion.

### Anaerobic digestion

A batch of anaerobic digestion experiments were conducted using anaerobic flasks with a working volume of 0.5 L. Three different treatments were set in this study, namely, ultrasonication-pretreated petrochemical sludge, thermal-pretreated petrochemical sludge, and fresh sludge (control), which were mixed with inoculated sludge at a volume ratio of 9:1 and then placed into the conical flasks, and no extra mineral and organic nutrient was added into the petrochemical sludge anaerobic digestion system. The inoculated sludge was collected from the anaerobic reactor of the Yangzi petrochemical wastewater treatment plant, with total solid content of 2.15% and volatile solids content of 30.2%. Oxygen in the conical flasks was removed by nitrogen gas sparging for 120 s, and then the flasks were capped with rubber stoppers and placed in an incubator (SPX-25013-D, China) at (38±1)°C. Gas sampling bags were used to collect the gas produced in each reactor. During the anaerobic digestion process, each conical flasks was shaken manually six times per day for 5 min to prevent the sludge from settling. During incubation, samples for chemical analysis were withdrawn at different digestion times.

### Analysis methods

The pH, total solid (TS), and volatile solid (VS) were measured according to the standard methods [19], and VS content is the percentage relative to the total solids. The dewaterability of sludge was measured as capillary suction time (CST) by using a capillary suction timer (Model 304M, Triton, Britain). Biogas production was analyzed by drainage method, and the methane content in the biogas samples was analyzed using a gas chromatograph (HP7890) equipped with thermal conductivity detector and PLOT-Q column (30 m-0.53 mm-15 l m). Helium was used as the carrier gas.

Sludge EPS was investigated using the ultrasonication-centrifugation method as described in detail previously [13]. The 0.45 μm acetate cellulose membranes and the dialysis membranes of MWCO of 3500 Da (Shanghai Sangon Biotechnology, China) were used to remove the particulates and low-molecular-weight metabolites in the slime, LB-EPS, and TB-EPS solutions before chemical analysis [13,21]. Protein content was measured by using the method proposed by Lowry et al. [22], and albumin was used as a standard solution [23]. Polysaccharide content in the EPS solution was measured by the anthrone method [24]. PN/PS was the content ratio of protein to polysaccharide. The total EPS was the sum of each layer of the sludge.

The procedure used to extract PAHs from the petrochemical sludge samples was according to Gao et al. [25]. Prior to use, all methods were tested for the efficiency of recovery. For PAHs amended sludge, recovery averaged 94% (n = 5, RSD less than 2.5%; RSD, relative standard deviation) for PAHs. The petrochemical sludge from the anaerobic digesters were carefully collected and freeze-dried, homogenized, and then passed through a 20-mesh standard sieve. Sample preparation included homogeneous mixing of 1 g of dried sludge sample with anhydrous Na_2_SO_4_ to remove moisture and ultrasonication in 10 mL dichloromethane for 1 h, followed by centrifugation. Then, 3 mL of supernatant was filtered through 2 g of silica gel column with 11 mL 1:1 (v/v) elution of hexane and dichloromethane. The solvent fractions were then evaporated and exchanged by methanol with a final volume of 2 mL, and then were analyzed with a high performance liquid chromatography (HPLC) fitted with a 4.6 x 150 mm reverse phase C18 column using methyl cyanide-water (4:6) as the mobile phase at a flow rate of 1.0 mL min^-1^, and were detected at 254 nm. Chromatography was performed at 30°C. All treatments were done in triplicate unless otherwise mentioned specifically.

### Statistical analysis

The data presented in the results section are the mean and standard deviation of triplicate samples collected and analyzed. The data were analyzed with SPSS (SPSS 20.0 for Windows). All of the figures presented include the standard deviations of the data and were drawn with Origin 8.5 software.

## Results and Discussion

### Biogas property of petrochemical sludge during anaerobic digestion

**Fig 1:**
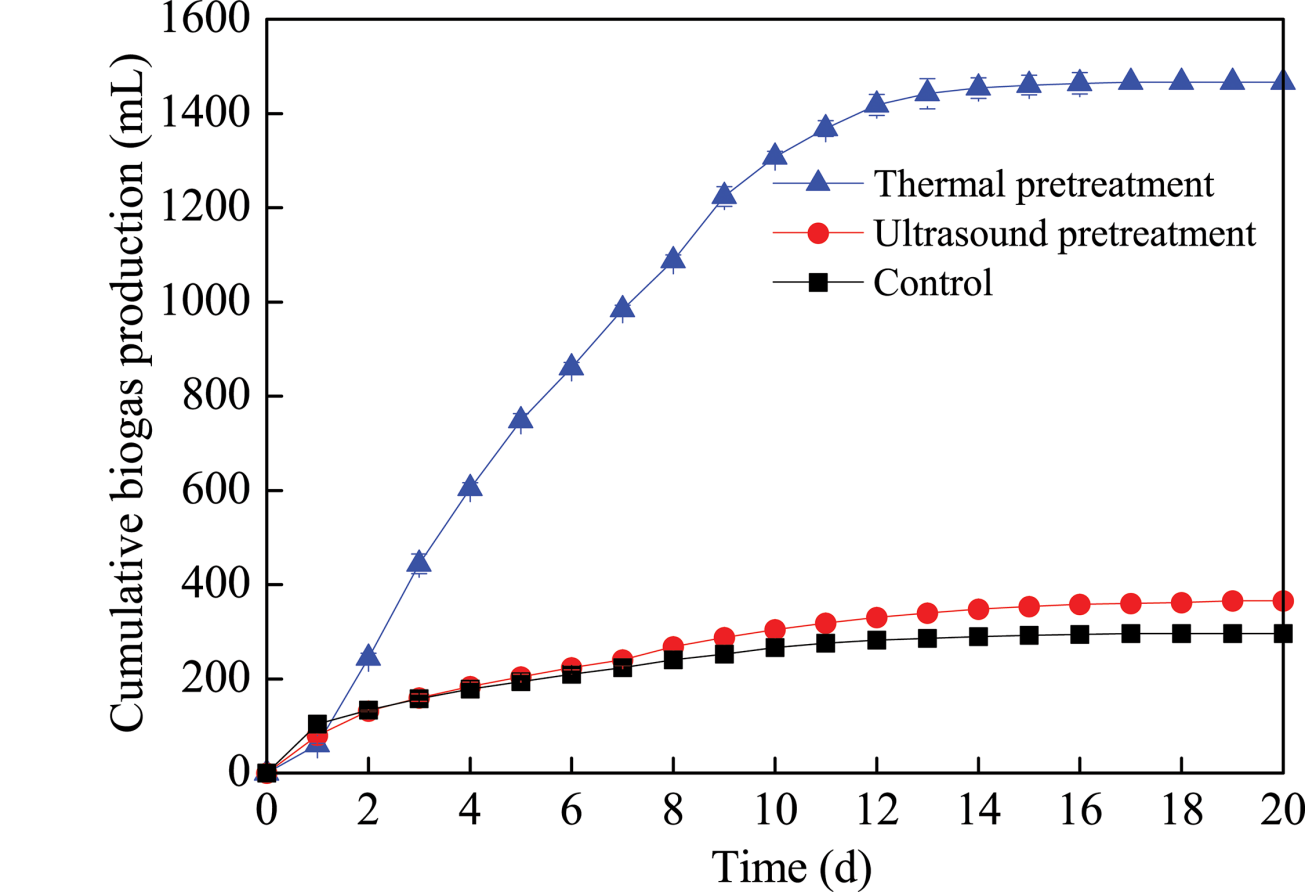
Cumulative biogas production of the petrochemical sludge during the anaerobic digestion.

The cumulative biogas production of the petrochemical sludge with different pretreatments during anaerobic digestion is shown in Fig 1. The cumulative biogas productions were 1466, 366 and 296 mL in the thermal pretreatment, ultrasound pretreatment, and the control system, respectively. The total biogas production volume in the thermal pretreatment system was 4 and 5 times higher than those in the ultrasound pretreatment and in the control system. Meanwhile, along with fermentation, biogas production of the three different treatments increased gradually and almost achieved their peaks in the first 12 days. No significant increase was observed after this period. The methane content in biogas is increased gradually, and the proportion of methane was remained constant and with values between 30%-50% for different condition. For thermal pretreatment system, the maximum methane content was 49.89%, while maximum methane content was just 30.23% for the control and ultrasound pretreatment system. Pretreatment can reportedly make more intracellular organic matter (protein, polysaccharide, etc.) release to the liquid phase of sludge and increase the SCOD [26], and then improve the hydrolysis rate of sludge anaerobic digestion. However, the methane content in biogas is lower than other reports [9,20], the reasons may be due to the differences in raw material. The pH is an important factor that influences the performance of anaerobic digestion, and the optimum pH range for effective decomposition of organic matter during anaerobic digestion is 6.3–7.8 [26]. The methanogenic activity will be inhibited at a pH less than 6.3 and higher than 7.8. The pH in the three treatments were all between 6.3 and 7.8, indicating healthy conditions inside the digester. A good amount of methanogenic activity was expected. A slight decrease in pH was noted among the three different treatments in the first 5 days, after which a slow increased was observed, suggesting that volatile fatty acids were produced as substrates for biogas production.

### VS reduction during petrochemical sludge anaerobic digestion using different pretreatment processes

**Fig 2:**
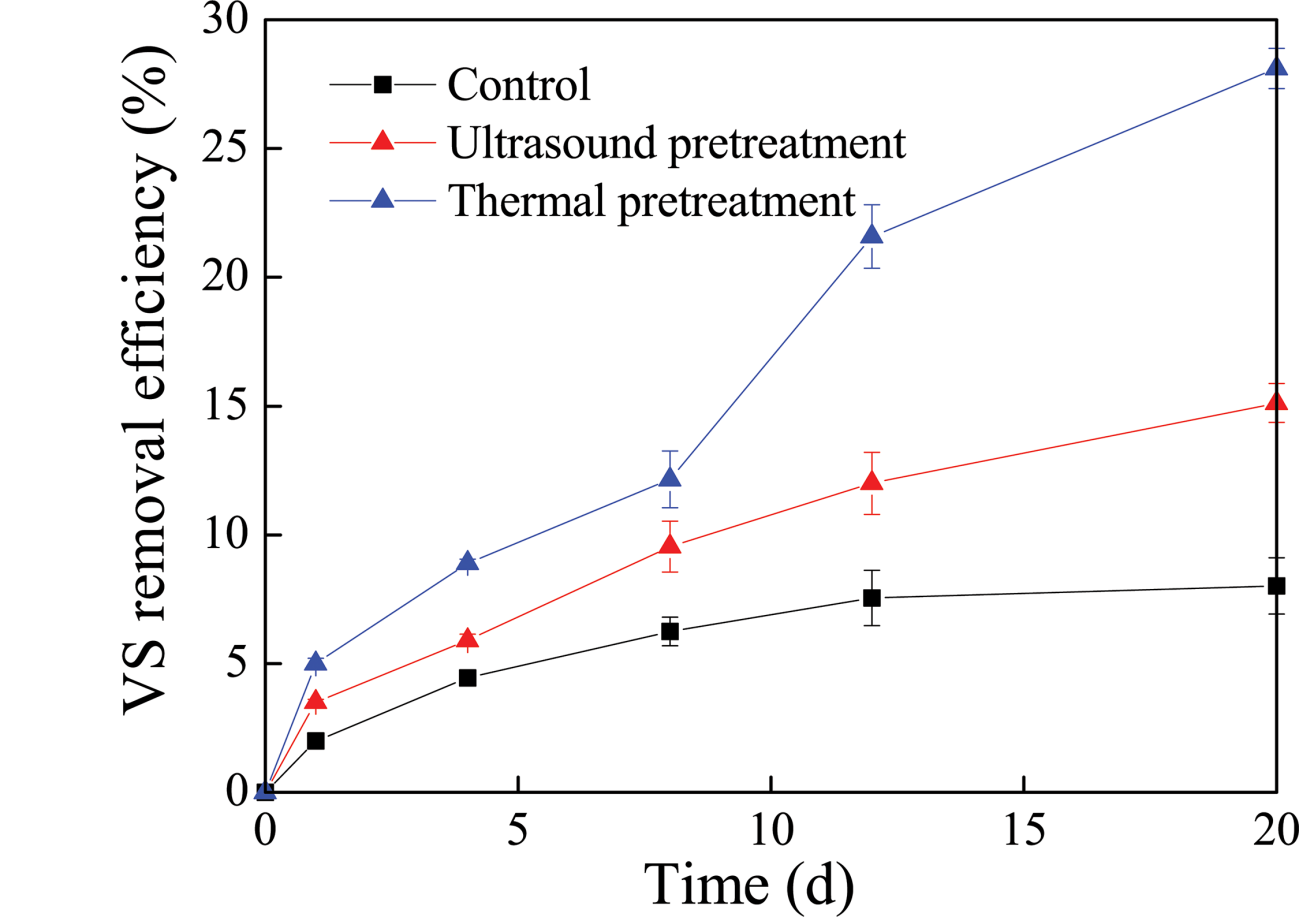
VS removal efficiency of different treatment during anaerobic digestion.

VS reduction is usually used to evaluate the reactor performance and stability of the digestate [6]. The variation of VS removal efficiency is presented in Fig 2. The overall performance of VS reduction efficiency increased with the sludge retention time. The VS removal efficiencies reached 28%, 15%, and 8% in the thermal pretreatment, ultrasound pretreatment, and the control system after anaerobic digestion, respectively. The highest performance was found in the thermal pretreatment system. It has been reported that thermal treatments could help break down the sludge microbial cells and facilitate the decomposition reaction, and make more intracellular organic matter (protein, polysaccharide, etc.) release to the liquid phase of sludge and increase the SCOD [26], and then lead to the biodegradation of more organic compounds in the digester [9]. Meanwhile, Guo et al. [20] showed that VS removal efficiency was reached 50% in the anaerobic co-digestion of thermal pre-treated municipal biowaste system, and more than 60% VS removal efficiency was achieved in the dairy waste activated sludge anaerobic digestion system reported by Rani et al. [26]. The differences of the VS removal efficiency may be due to the differences in raw materials.

### Variation of sludge dewaterability during petrochemical sludge anaerobic digestion

**Fig 3:**
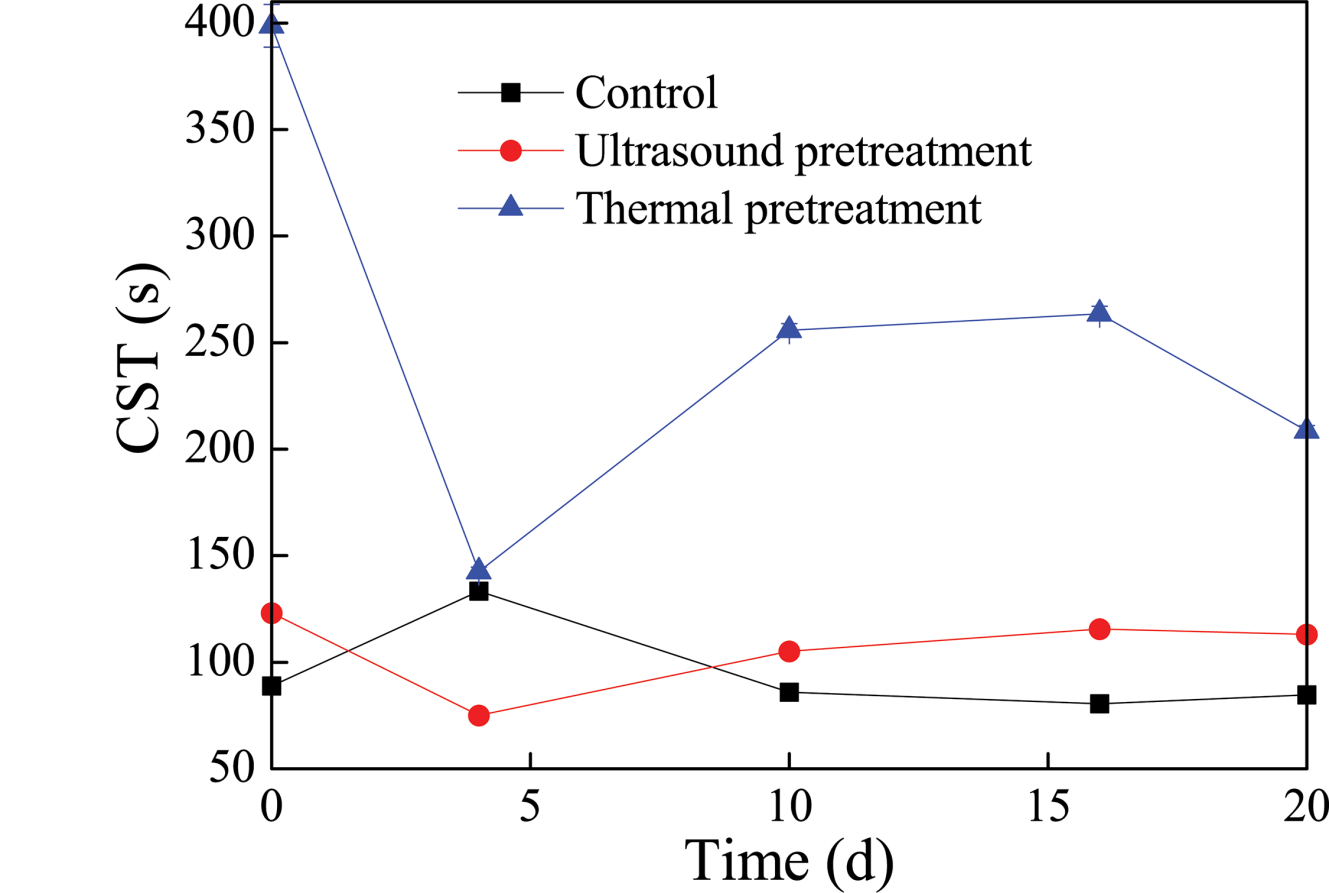
Changes of sludge CST during the petrochemical sludge anaerobic digestion.

Sludge CST is a widely used means of gauging sludge dewaterability [25–26]. It was found from the Fig 3 that sludge CST increased to 398.7 and 123.1 s after thermal and ultrasound pretreatment, which was more than that of the control (88.9 s). The increment of sludge CST after pretreatment may be due to the change of EPS distribution in the sludge [13,27,28]. Sludge CST gradually increased in the control system and then exhibited a slight decrease at the end of anaerobic digestion, indicating that the dewaterability of sludge improved slightly after anaerobic digestion. In the thermal pretreatment system, sludge CST was shortened from 398.7 s to 142.5 s in 4 days, and then gradually increased. The dewaterability of sludge in the ultrasound pretreatment system was similar to that of the thermal pretreatment system, as shown by the sludge CST in the ultrasound system that decreased from 123.1 s to only 75.1 s in the first 4 days, and then gradually increased to 113.1 s at the end of the anaerobic digestion process. These results indicated that the dewaterability of sludge was improved by the anaerobic digestion process, and exhibited a little deterioration with the prolonged anaerobic digestion time.

### Pearson correlation analyses between sludge CST and protein, polysaccharide, and PN/PS in every layer of sludge flocs

EPS is regarded as the key factor in regulating sludge dewaterability [11–12]. Thus, the sludge dewaterability during anaerobic digestion may result from the variation of sludge EPS [18,29]. While their respective contribution on the sludge dewaterability enhancement was still unclear. Therefore, pearson correlation analysis between sludge CST and the content of protein, polysaccharide, and PN/PS ratio in each layer of sludge was applied here to analyze their respective contribution on the sludge dewaterability, and results are shown in Table 2. Findings indicated that sludge CST was correlated with protein content (R = 0.74653, p = 0.00139) and polysaccharide content (R = 0.7691, p = 0.0008) in the slime-EPS of sludge flocs, whereas it had no correlation with PN/PS (R = 0.06436, p = 0.8975) in the slime layer. Protein and polysaccharide contents in the control slowly increased in the anaerobic digestion process, while the amount of protein in slime sharply decreased in the first 4 days, and then fluctuated within the range of 2–4 mg/g SS. Meanwhile, polysaccharide content in the slime of ultrasound and thermal pretreatment systems decreased during the whole anaerobic digestion (S1 Fig). Thus, it can be concluded that protein and polysaccharide in slime layer had an important role in the sludge dewaterability, and the increase of protein and polysaccharide content would lead to the deterioration of sludge dewaterability. On the contrary, the decrease of protein content in the slime layer would improve the dewaterability of sludge. And this phenomenon was also found in the bioleaching system as described by Zhou et al. [13].

**Table 2:** Pearson correlations between sludge CST and the content of protein (a), polysaccharide (b) and PN/PS (c) from Slime, LB, TB, Slime+LB+TB layer of sludge in the sludge anaerobic digestion systems.

Parameters	Protein	polysaccharide	PN/PS
Slime	R = 0.74653, p = 0.0014 (+)	R = 0.7691,P = 0.0008 (×)	R = 0.06436, p = 0.8975 (×)
LB-EPS	R = 0.90257, p<0.0001 (+)	R = 0.86941, p<0.0001 (+)	R = -0.08364 p = 0.76697 (×)
TB-EPS	R = 0.89491, p<0.0001 (+)	R = 0.4374, p = 0.1030 (×)	R = 0.20773, p = 0.45754(×)
Total EPS	R = 0.91116, p<0.0001 (+)	R = 0.93916, p<0.0001 (+)	R = -0.15012, p = 0.59333 (×)

+ Positive correlation; × no correlation

Similar results were also found in the LB-EPS, and it was found that sludge CST was also correlated with the content of protein (R = 0.90257, p<0.0001) and polysaccharide (R = 0.86941, p<0.0001) in the LB-EPS layer. Results in S2 Fig showed that protein and polysaccharide contents in the LB-EPS of the three different treatments all decreased initially and then gradually increased. Therefore, the decrease of protein and polysaccharide contents LB-EPS were responsible for sludge dewaterability enhancement in the anaerobic digestion. In the TB-EPS layer, sludge CST was correlated with the content of protein (R = 0.89491, p<0.0001) but had no correlation with polysaccharide (R = 0.4374, p = 0.103) and PN/PS (R = 0.20773, p = 0.45754). Changes in the chemical compositions of TB-EPS during sludge anaerobic digestion were presented in S3 Fig. Protein and polysaccharide contents in TB-EPS gradually decreased in all these systems, and then increased slowly during the anaerobic digestion. Therefore, the increase of protein in the TB-EPS layer would lead to the deterioration of sludge dewaterability. Sludge CST was correlated with protein content (R = 0.91116, p<0.0001) and polysaccharide content (R = 0.93916, p<0.0001) of the total EPS, whereas it had no correlation with PN/PS (R = -0.15012, p = 0.59333) of the total EPS. It can be seen that the total protein and polysaccharide contents were increased to 3.44 and 2.13 mg/g SS after ultrasound pretreatment, and the total protein and polysaccharide contents increased to 19.36 and 8.80 mg/g SS after thermal pretreatment pretreatment (S4 Fig). Obviously, the pretreatments enhanced the petrochemical sludge solubilization, and then increased the likelihood of the degradation of the sludge. The total protein and polysaccharide contents in all the sludge layers gradually decreased in all these systems in the first 4 days, and then increased slowly during the anaerobic digestion. An analysis of the stratification in this study showed that the decrease of protein and polysaccharide contents in the slime, LB, and Slime+LB+TB layers, and the decreases of protein content in TB-EPS were responsible for sludge dewaterability enhancement in the anaerobic digestion system. Neyens et al. [16] concluded that the advanced methods could degrade proteins and polysaccharides of sludge EPS, and then reduce their water retention properties thereby releasing the EPS-bound water and increasing the dewatering efficiency of sludge. Results in this study was similar with Neyens et al. [16].

### PAHs biodegradation efficiency during petrochemical sludge anaerobic digestion

**Fig 4:**
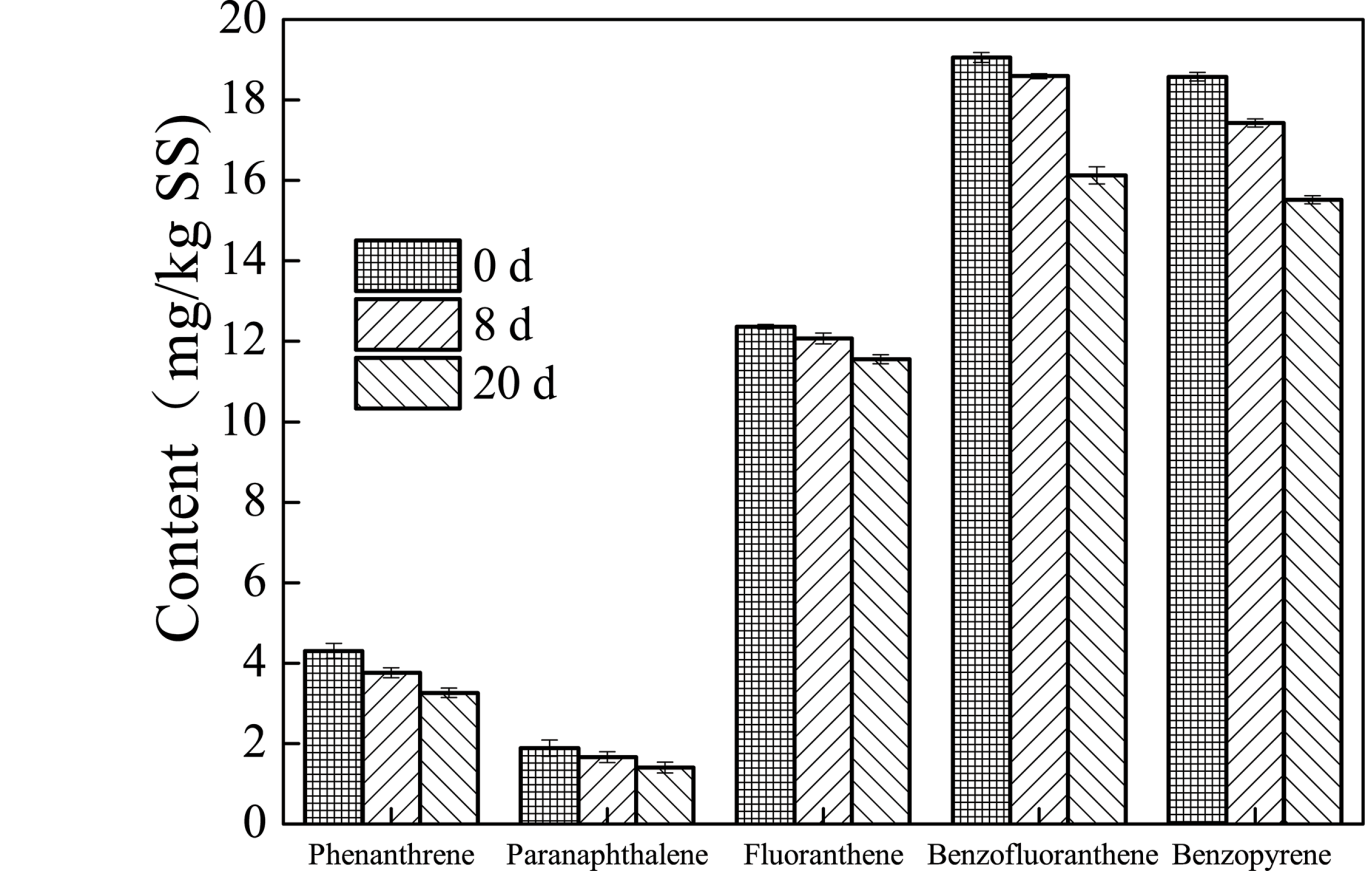
Variation of the content of PAHs during petrochemical sludge anaerobic digestion in the control system.

**Fig 5:**
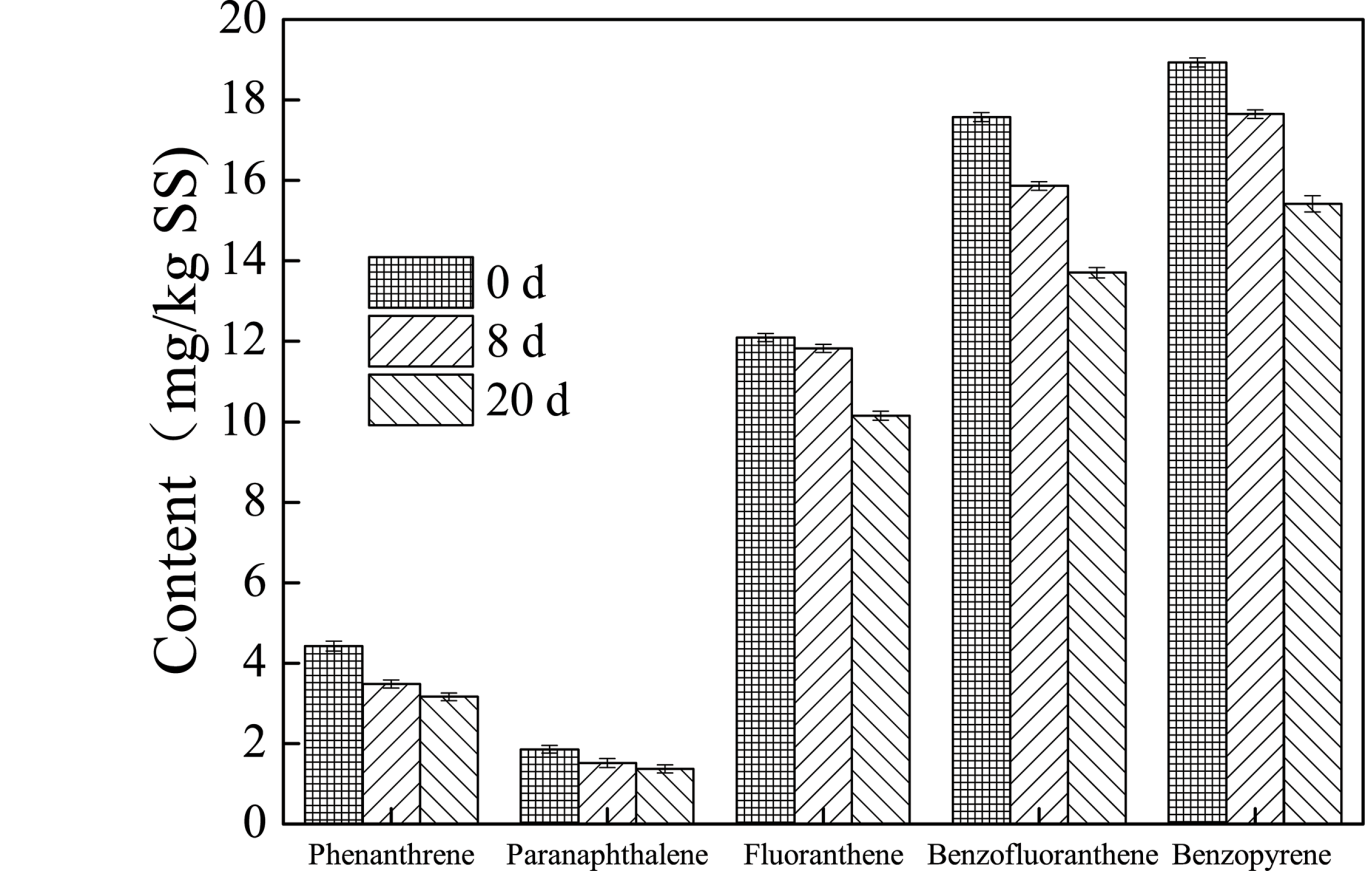
Variation of the content of PAHs during petrochemical sludge anaerobic digestion in the ultrasound pretreatment system.

**Fig 6:**
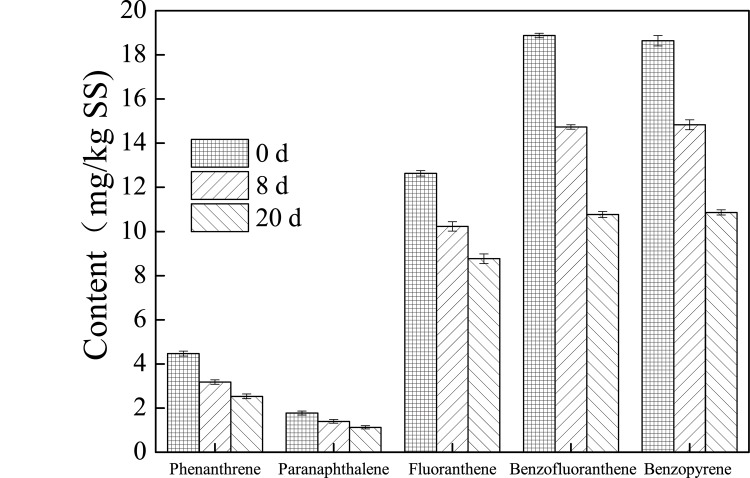
Variation of the content of PAHs during petrochemical sludge anaerobic digestion in the thermal pretreatment system.

PAHs pollution is a limiting factor in the subsequent treatment of petrochemical sludge, so elucidating the variation of PAHs during the sludge anaerobic digestion is important. The contents of PAHs during the anaerobic digestion with different pretreatments are shown in Figs 4, 5 and 6. The contents of phenanthrene, paranaphthalene, fluoranthene, benzofluoranthene, and benzopyrene were 4.4678, 1.775, 12.632, 18.876, and 18.644 mg/kg TS in the original petrochemical sludge. The content of PAHs in the petrochemical sludge decreased during the anaerobic digestion. It was found that phenanthrene, paranaphthalene, fluoranthene, benzofluoranthene, and benzopyrene contents were 2.532, 1.126, 8.7655, 10.765, and 10.863 mg/Kg TS at the end of 20 days of anaerobic digestion in the thermal pretreatment system. The removal rates in the thermal pretreatment system reached 43.3%, 55.5%, 30.6%, 42.9%, and 41.7%, repectively, which were much higher than those in the ultrasound pretreatment (28.5%, 25.9%, 16.0%, 22.0%, 18.5%) and the control system (24.2%, 25.5%, 6.58%, 15.4%, 16.4%). This observation proves that pretreatment not only helps improve biogas production, but also increases the removal rate of PAHs. It has been reported increase in PAHs biodegradation is caused by either transfer of PAHs from sorption sites with low desorption rates to those with high ones or transformation of slow-sorption sites into fast-sorption ones through thermal pretreatment [30]. In addition, pretreatment could help break down the sludge microbial cells and facilitate the decomposition reaction, and make more intracellular organic matter (protein, polysaccharide, etc.) release to the liquid phase of sludge (S1–S4 Figs) and increase the SCOD, and the SCOD maybe as cometabolism substrate for the biodegradation of more PAHs in the digester [31–32]. This phenomenon was also found in other studies [33–35]. Bernal-Martinez et al. [33–34] found that the PAH removal rate was about 20%-70%, and ozonation pre-treatment could led to the enhancement of PAH biodegradability through the enhancement of bioavailability, and Barret et al. [35] found that the total removal rate of low molecular weight PAHs reached 25%. However, a large amount of PAHs still remain in the sludge after anaerobic digestion. To enhance the mass transfer, and thus, PAH removals, various operating conditions, such as increasing the temperature and adding surfactant or methanol, need to be tested in future studies [36].

## Conclusion

The total biogas production volume in thermal pretreatment system was 4 and 5 times higher than those in ultrasound pretreatment and the control system. The corresponding VS removal efficiencies reached to 28.1%, 15.1%, and 8.02%. Phenanthrene, paranaphthalene, fluoranthene, benzofluoranthene and benzopyrene removal rates were 43.3%, 55.5%, 30.6%, 42.9%, and 41.7% in thermal pretreatment system. The decrease of protein and polysaccharide in the sludge could improve sludge dewaterability during petrochemical sludge anaerobic digestion. These findings indicated that thermal pretreatment might be a promising enhancement method for the degradation of PAHs, biogas production, and improvement of dewaterability during petrochemical sludge anaerobic digestion.

## Supporting Information

S1 Fig Variation of the content of protein (a), polysaccharide (b) and PN/PS (c) in the slime during sludge anaerobic digestion process (PN/PS: protein/ polysaccharide). (DOC)

S2 Fig Variation of the content of protein (a), polysaccharide (b) and PN/PS (c) in the LB-EPS during sludge anaerobic digestion process (PN/PS: protein/ polysaccharide). (DOC)

S3 Fig Variation of the content of protein (a), polysaccharide (b) and PN/PS (c) in the TB-EPS during sludge anaerobic digestion process (PN/PS: protein/ polysaccharide). (DOC)

S4 Fig Variation of the content of protein (a), polysaccharide (b) and PN/PS (c) in the total EPS during sludge anaerobic digestion process (PN/PS: protein/ polysaccharide). (DOC)

## References

[1] Appels L, Baeyens J, Degreve J, Dewil R (2008) Principles and potential of the anaerobic digestion of waste-activated sludge. Prog Energy Combust Sci 34: 755–781.

[2] Ma XY, Duan YF, Liu M (2013) Effects of petrochemical sludge on the slurry-ability of coke water slurry. Exp Thermal Fluid Sci 48: 238–244.

[3] Chen JL, Ortiz R, Steele TWJ, Stuckey DC (2014) Toxicants inhibiting anaerobic digestion: a review. Biotechnol Adv 32: 1523–1534. 10.1016/j.biotechadv.2014.10.005 25457225

[4] Ruiz B, Flotats X (2014) Citrus essential oils and their influence on the anaerobic digestion process: an overview. Waste Manage 34: 2063–2079. 10.1016/j.wasman.2014.06.026 25081855

[5] Tiehm A, Nickel K, Zellhorn M, Neis U (2001) Ultrasonic waste activated sludge disintegration for improving anaerobic stabilization. Water Res 35: 2003–2009. 11337847 10.1016/s0043-1354(00)00468-1

[6] Pilli S, Bhunia P, Yan S, LeBlanc RJ, Tyagi RD, Surampalli RY (2011) Ultrasonic pretreatment of sludge: A review. Ultrason Sonochem 18: 1–18. 10.1016/j.ultsonch.2010.02.014 20471901

[7] Uma Rani R, Kaliappan S, Adish Kumar S, Rajesh Banu J (2012) Combined treatment of alkaline and disperser for improving solubilization and anaerobic biodegradability of dairy waste activated sludge. Bioresour Technol 126: 107–116. 10.1016/j.biortech.2012.09.027 23073096

[8] Yeom IT, Lee KR, Lee YH, Ahn KH, Lee SH (2002) Effects of ozone treatment on the biodegradability of sludge from municipal wastewater treatment plants. Water Sci Technol 46: 421–425. 12361042

[9] Bougrier C, Delgenès JP, Carrère H (2008) Effects of thermal treatments on five different waste activated sludge samples solubilisation, physical properties and anaerobic digestion. Chem Eng J 139: 236–244.

[10] Burgess JE, Pletschke BI (2008) Hydrolytic enzymes in sewage sludge treatment: a mini-review. Water Sa 34: 343–349.

[11] Li XY, Yang SF (2007) Influence of loosely bound extracellular polymeric substances (EPS) on the flocculation, sedimentation and dewaterability of activated sludge. Water Res 41: 1022–1030. 16952388 10.1016/j.watres.2006.06.037

[12] Liu XM, Sheng GP, Luo HW, Zhang F, Yuan SJ, Xu J, et al (2010) Contribution of extracellular polymeric substances (EPS) to the sludge aggregation. Environ Sci Technol 44: 4355–4360. 10.1021/es9016766 20446688

[13] Zhou J, Zheng GY, Zhang XY, Zhou LX (2014) Extracellular Polymeric Substances Influence on the Dewaterability of Sewage Sludge during Bioleaching. Plos one 9, e102688 10.1371/journal.pone.0102688 25050971 PMC4106846

[14] Houghton JI, Quarmby J, Stephenson T (2001) Municipal wastewater sludge dewaterability and the presence of microbial extracellular polymer. Water Sci Technol 44: 373–379. 11548008

[15] Yu GH, He PJ, Shao LM, He PP (2008) Stratification structure of sludge flocs with implications to dewaterability. Environ Sci Technol 42: 7944–7949. 19031885 10.1021/es8016717

[16] Neyens E, Baeyens J, Dewil R, Deheyder B (2004) Advanced sludge treatment affects extracellular polymeric substances to improve activated sludge dewatering. J Hazard Mater 106: 83–92. 15177096 10.1016/j.jhazmat.2003.11.014

[17] Mahmoud A, Olivier J, Vaxelaire J, Hoadley AFA (2011) Electro-dewatering of wastewater sludge: influence of the operating conditions and their interactions effects. Water Res 45: 2795–2810. 10.1016/j.watres.2011.02.029 21453949

[18] Yu GH, He PJ, Shao LM (2010b) Reconsideration of anaerobic fermentation from excess sludge at pH 10.0 as an eco-friendly process. J Hazard Mater 175: 510–517. 10.1016/j.jhazmat.2009.10.035 19896767

[19] APHA, AWWA, WEF (2005) Standard methods for the examination of water and wastewater, 21th ed., American Public Health Association, Washington DC.

[20] Guo JB, Wang W, Liu X, Lian SJ, Zheng L (2014) Effects of thermal pre-treatment on anaerobic co-digestion of municipal biowastes at high organic loading rate. Chemosphere 101: 66–70. 10.1016/j.chemosphere.2013.12.007 24374189

[21] Yu GH, He PJ, Shao LM, Lee DJ (2007) Enzyme activities in activated sludge flocs. Appl Microbiol Biotechnol 77: 605–612. 17932668 10.1007/s00253-007-1204-5

[22] Lowry OH, Rosebrough NJ, Farn A, Randall R (1951) Protein measurement with the folin phenol reagent. J Biol Chem 193: 265–275. 14907713

[23] Frølund B, Keiding K, Nielsen P (1995) Enzymatic activity in the activated sludge sludge flocs matrix. Appl Microbiol Biotechnol 43: 755–761. 7546613 10.1007/BF00164784

[24] Gaudy AF (1962) Colorimetric determination of protein and carbohydrate. Ind Water Wastes 7: 17–22.

[25] Gao YZ, Zhu LZ (2004) Plant uptake, accumulation and translocation of phenanthrene and pyrene in soils. Chemosphere 55: 1169–1179. 15081757 10.1016/j.chemosphere.2004.01.037

[26] Rani RU, Kumar SA, Kaliappan S, Yeom IT, Banu JR (2014) Enhancing the anaerobic digestion potential of dairy waste activated sludge by two step sono-alkalization pretreatment. Ultrason Sonochem 21: 1065–1074. 10.1016/j.ultsonch.2013.11.007 24309086

[27] Chen Y, Yang H, Gu G (2001) Effect of acid and surfactant treatment on activated sludge dewatering and setting. Water Res 5: 2615–2620. 10.1016/s0043-1354(00)00565-0 11456159

[28] Yu GH, He PJ, Shao LM (2010a) Novel insights into sludge dewaterability by fluorescence excitation–emission matrix combined with parallel factor analysis. Water Res 44: 797–806. 19914675 10.1016/j.watres.2009.10.021

[29] Novak JT, Sadler ME, Murthy SN (2003) Mechanisms of floc destruction during anaerobic and aerobic digestion and the effect on conditioning and dewatering of biosolids. Water Res 37: 3136–3144. 14509700 10.1016/S0043-1354(03)00171-4

[30] Bonten LTC, Grotenhuis TC, Rulkens WH (1999) Enhancement of PAH Biodegradation in soil by physicochemical pretreatment. Chemosphere 38: 3627–3636. 10365437 10.1016/s0045-6535(98)00574-8

[31] Znila A (2013) Update on the cometabolism of organic pollutants by bacteria. Environ Pollut 178: 474–482. 10.1016/j.envpol.2013.03.042 23570949

[32] Haritash AK, Kaushik CP (2009) Biodegradation aspects of Polycyclic Aromatic Hydrocarbons (PAHs): A review. J Hazard Mater 169: 1–15. 10.1016/j.jhazmat.2009.03.137 19442441

[33] Bernal-Martinez A, Carrere H, Patureau D, Delgenes JP (2005) Combining anaerobic digestion and ozonation to remove PAH from urban sludge. Process Biochem 40: 3244–3250.

[34] Bernal-Martinez A, Carrere H, Patureau D, Delgenes JP (2007) Ozone pre-treatment as improver of PAH removal during anaerobic digestion of urban sludge. Chemosphere 68: 1013–1019. 17382369 10.1016/j.chemosphere.2007.02.019

[35] Barret M, Carrere H, Delgadillo L, Patureau D (2010) PAH fate during the anaerobic digestion of contaminated sludge: Do bioavailability and/or cometabolism limit their biodegradation? Water Res 44: 3797–3806. 10.1016/j.watres.2010.04.031 20569963

[36] Barret M, Mirquez LD, Trably E, Delgenes S N, Braun F, Barcia GC, et al (2012) Anaerobic removal of trace organic contaminants in sewage sludge: 15 years of experience. Pedosphere 22: 508–517.

