# Supplementary material for: Ultrasonic and Thermal Pretreatments on Anaerobic Digestion of Petrochemical Sludge: Dewaterability and Degradation of PAHs
Source: PLoS One. 2015 Sep 1;10(9):e0136162. doi: 10.1371/journal.pone.0136162 (PMC4556613; doi:10.1371/journal.pone.0136162)
Supplement: S3 Fig — (DOC) [file pone.0136162.s003.doc]

**Figure S3:** Variation of the content of protein (a), polysaccharide (b) and PN/PS (c) in the TB-EPS during sludge anaerobic digestion process (PN/PS: protein/ polysaccharide)
